# Beyond mindfulness: the importance of body compassion in colorectal cancer distress

**DOI:** 10.3389/fpsyg.2025.1618389

**Published:** 2025-10-08

**Authors:** Lauren A. Zimmaro, Aimee J. Christie, Andrew Nicklawsky, Jennifer K. Altman, James W. Carson, Christopher H. Lieu, Carolyn Y. Fang, Jennifer B. Reese

**Affiliations:** ^1^Division of Medical Oncology, Department of Medicine, University of Colorado School of Medicine, University of Colorado Anschutz Medical Campus, Aurora, CO, United States; ^2^University of Colorado Cancer Center, University of Colorado Anschutz Medical Campus, Aurora, CO, United States; ^3^Department of Palliative, Rehabilitation, and Integrative Medicine, The University of Texas MD Anderson Cancer Center, Houston, TX, United States; ^4^Department of Rehabilitation Medicine, University of Washington, Seattle, WA, United States; ^5^Departments of Anesthesiology and Perioperative Medicine, and Psychiatry, Oregon Health & Science University, Portland, OR, United States; ^6^Cancer Prevention and Control Program, Fox Chase Cancer Center, Philadelphia, PA, United States

**Keywords:** colorectal cancer, distress, mindfulness, body compassion, coping

## Abstract

**Objective:**

Psychological distress is common among people diagnosed with colorectal cancer (CRC), often stemming from physical changes and challenges associated with the disease and its treatment. Body compassion, a mindfulness-related construct emphasizing acceptance, defusion, and common humanity of the physical body, may offer new perspectives on the link between mindfulness and distress in cancer patients, but this remains unexplored. This study investigated the relationship between mindfulness, body compassion, and distress in individuals with CRC.

**Methods:**

Fifty-four people diagnosed with CRC completed surveys assessing demographic and medical characteristics [e.g., stage, treatment status, medical comorbidities (SCQ: Self-Administered Comorbidity Questionnaire)], mindfulness (FFMQ-15), body compassion (BCS), and distress (HADS). Relationships were assessed with Holm-corrected Pearson's correlations. Regression models of mindfulness and distress explored a potential mediating effect of body compassion. Interactions between body compassion and disease burden variables (e.g., SCQ) were explored for moderation.

**Results:**

Mindfulness and body compassion were moderately correlated (*r* = 0.62, *p* < 0.001), with the strongest relationships observed between the subscales of mindful non-judgment and body compassion defusion (*r* = 0.70, *p* < 0.05). Greater mindfulness was associated with lower distress (*B* = −0.39, CI_95%_ [−0.56, −0.16], *p* < 0.001). This relationship was significantly mediated by body compassion (*B* = −0.21, CI_95%_ [−0.38, −0.08], *p* < 0.001), which accounted for 54% of the total effect (*p* < 0.001). No evidence of moderation was observed.

**Conclusions:**

Among individuals with CRC, body compassion appears to be a key factor within the mindfulness-distress relationship. Future studies are warranted, particularly experimental designs to assess body compassion as a potential mechanism by which mindfulness-based interventions improve distress.

## Introduction

Psychological distress is prevalent in people diagnosed with colorectal cancer (CRC), with symptoms of depression and anxiety endorsed in up to 57% and 47% of the CRC population, respectively (Peng et al., 2019). As evidenced by both quantitative and qualitative data, distress among individuals with CRC often stems from difficulty coping with changes to the physical body, both in image and function, as a result of the disease and/or its treatment (Khoo et al., 2022; Kovoor et al., 2023; Phung and Fang, 2023; González et al., 2016; Lim et al., 2021; Mohamed et al., 2021). Recent reviews and meta-analyses indicate that the most frequent, severe, and distressing symptoms reported by people with CRC are indeed centered on changes in their physical bodies; these symptoms include disruptions in gastrointestinal and bowel function, body image distress, sexual dysfunction, peripheral neuropathy, fatigue, and insomnia (Phung and Fang, 2023; Averyt and Nishimoto, 2014; Han et al., 2020; Waddell et al., 2023). These findings are reinforced by research demonstrating that CRC patients with greater disease burden—such as those with metastatic disease, ostomies, or medical comorbidities, those diagnosed at younger ages, or those undergoing active treatment—frequently experience higher levels of distress compared to those with less severe disease (Kovoor et al., 2023; Han et al., 2020; Waddell et al., 2023; Potosky et al., 2022; Petrova et al., 2021). Collectively, these findings suggest that when coping with CRC, a patient's relationship with their physical body—specifically, their disease burden and their ability to manage physical symptoms—is highly relevant to their distress and quality of life.

When coping with cancer, personal psychological resources play an important role in how individuals navigate disease-related challenges. One such resource is mindfulness, which is defined as intentional and non-evaluative awareness of the present moment, focusing on maintaining non-judgmental awareness and acceptance of present-moment experiences, including thoughts, emotions, sensations, and environmental surroundings (Kabat-Zinn, 2009). Indeed, mindfulness has gained substantial scientific and clinical support as a mechanism for promoting individuals' adjustment to cancer (Heinen et al., 2024). Extensive quantitative and qualitative research demonstrates that mindfulness (whether as a dispositional trait or learned coping skill) is effective for reducing distress, including symptoms of anxiety and depression, among cancer patients and survivors (Heinen et al., 2024; Wang et al., 2024; Fan et al., 2023; McCloy et al., 2022; Chayadi et al., 2022; Pedro et al., 2021; Xunlin et al., 2020; Cillessen et al., 2019; Zhou et al., 2024). While mindfulness is traditionally taught as a broad coping skill—intended to be applied flexibly across a wide range of present-moment experiences (Kabat-Zinn and Hanh, 2009)—there is growing recognition that more targeted mindfulness interventions and/or skills may be needed to fully address the specific and evolving psychosocial needs of individuals, including those coping with cancer (Zhou et al., 2024; Loucks et al., 2022; Zimmermann et al., 2018). In the context of colorectal cancer, one important step in this direction is clarifying the relationship between dispositional mindfulness (a general tendency toward present-moment awareness) and more nuanced mindfulness constructs, such as mindful awareness of the body. Clarifying this distinction is essential for developing more responsive mindfulness-based interventions that are aligned with the realities of patients' disease experiences and treatment trajectories.

Body compassion, a construct related to mindfulness, offers one such avenue for exploration, given its particular relevance for coping with distress related to the physical changes of cancer and its treatment (Zimmaro et al., 2024). Body compassion is a multidimensional construct that focuses on fostering a compassionate stance toward one's own physical body (Altman et al., 2017). Three factors contribute to the construct of body compassion: (1) defusion [recognizing that the physical body is just one aspect of the self and that maladaptive thoughts about the body (i.e., criticism, judgment) are transient mental experiences rather than absolute truths], (2) common humanity [acknowledging body-related challenges as a natural part of the human experience, rather than viewing them through a lens of shame or feeling isolated because of them], and (3) acceptance [responding to body-related painful thoughts and feelings with kindness and understanding, rather than with judgment or criticism] (Altman et al., 2017). Body compassion applies mindfulness and acceptance skills directly to the physical body, fostering openness, non-judgment, and compassionate curiosity (Altman et al., 2017, 2020).

Previous research on body compassion has largely centered on eating pathology and body dissatisfaction, with studies showing an inverse relationship between body compassion and those outcomes (Policardo et al., 2022; Oliveira et al., 2018; Barata-Santos et al., 2019; Khanjani et al., 2025; de Carvalho Barreto et al., 2020). In other studies of women in the general population, higher body compassion has been correlated with higher psychological wellbeing and positive affect (Altman et al., 2020; Policardo et al., 2022; Van Niekerk et al., 2022), as well as lower psychological inflexibility and negative affect (Altman et al., 2020; Policardo et al., 2022; Van Niekerk et al., 2022, 2023). In a sample of women with polycystic ovary syndrome, body compassion was positively associated with higher levels of perceived physical health (Van Niekerk et al., 2022). Interestingly, one study of Portuguese women in the general population found evidence to suggest that body compassion buffers body image-focused shame, or the perception that one's body is inferior, unattractive, undesirable, and vulnerable to criticism or rejection (Oliveira et al., 2018). These findings highlight the potential relevance of body compassion for coping with physical and emotional sequelae of illnesses, such as colorectal cancer.

An Italian research group has recently explored the role of body compassion as it relates to body image in breast cancer survivors. Sebri et al. (2022) developed a four-session body compassion intervention to help manage negative emotions and poor body image after breast cancer; early pilot study results showed reductions in anxiety, but not depression or improvements in body image (Sebri et al., 2023). This work has primarily focused on the theoretical overlap between body compassion and self-compassion with the aim of addressing body-image related distress. The present study examines body compassion from a distinct perspective, specifically investigating the overlap between body compassion and mindfulness, as well as their relationship to overall distress. Furthermore, examining these relationships in CRC survivors has particular clinical relevance due to the aforementioned changes in the physical body (Van Niekerk et al., 2022, 2023).

We recently published the first study to date that explored body compassion in cancer patients, which provided a preliminary description of the construct and its psychosocial correlates in CRC (Zimmaro et al., 2024). Results showed that body compassion was lower in those with metastatic disease and those in treatment (Zimmaro et al., 2024). Furthermore, higher body compassion was associated with lower distress and loneliness, and higher resilience and quality of life in CRC (Zimmaro et al., 2024). These findings underscore the need to investigate the role of body compassion in the psychosocial processes of cancer adjustment. Because mindfulness is an evidence-based mechanism for reducing distress (Heinen et al., 2024), examining body compassion as a potential complementary factor within the mindfulness–distress relationship represents a logical and timely next step. When distress relates to changes to the physical body (such as in CRC), it seems reasonable that body compassion (i.e., a targeted coping skill) might be more relevant than generalized mindfulness due to the shared underpinnings of distress and body compassion being centered on the physical body. Yet, the distinct relationship between mindfulness and body compassion—and their unique contributions to distress outcomes among cancer patients—remains unexplored. To address this gap, we conducted secondary exploratory analyses among that dataset.

We conducted an investigation of mindfulness, body compassion, and distress via a secondary analysis of cross-sectional survey data from a sample of CRC survivors (Zimmaro et al., 2024). Our aims were to (1) characterize the association between mindfulness and body compassion, (2) explore the degree to which body compassion plays a role in the well-established link between mindfulness and distress, and (3) examine whether disease burden affects the relationship between body compassion and distress. Although exploratory, we hypothesized (1) that body compassion and mindfulness would be moderately and positively correlated, reflecting that the constructs are related but not redundant; (2) that body compassion would help explain the relationship between mindfulness and distress among individuals with CRC; and (3) that the role of body compassion in the mindfulness-distress model would be moderated by disease burden.

## Methods

### Participants and procedures

Participants were recruited as part of an observational cross-sectional study examining distress, resilience, and coping among people with CRC, with sample size determination based upon a primary outcome of distress; the present study is a secondary analysis from that dataset. Our recently published manuscript (Zimmaro et al., 2024) details the methodology, which we summarize below for clarity. The study was approved by the IRB (Protocol #20-8018), and all participants provided informed consent before enrollment. Participants were recruited between May 2021 and August 2022 from an NCI-designated Comprehensive Cancer Center in Philadelphia, PA.

Cancer registry databases and clinic schedules were reviewed to identify individuals who had received a diagnosis of stage I-IV colon or rectal cancer within the past 5 years (new or recurrent disease), were 18 years of age or older, and were English speaking. Individuals who had significant cognitive (e.g., dementia), psychiatric (e.g., active symptoms of psychosis, poorly controlled serious mental illness), or medical conditions (e.g., life expectancy less than 6 months, in hospice care, etc.) were deemed ineligible. Eligible patients were approved for contact by their oncologist before being sent a study introduction email and/or letter (*n* = 200 eligible) and contacted by study staff for informed consent procedures (*n* = 72 could be reached; 18 of which declined participation). A total of 54 participants consented to participate and completed online study surveys via REDCap. Participants were provided with a $20 gift card upon survey completion.

### Measures

#### Demographic and medical variables

Demographics were obtained through a survey that patients completed at enrollment. Medical characteristics (age at CRC diagnosis, disease stage, metastatic disease status, current treatment status, and history of ostomy) were identified through medical record review.

#### Medical comorbidities

Medical comorbidities were measured using the Self-Administered Comorbidities Questionnaire (SCQ), which is a brief and comprehensive questionnaire that allows participants to evaluate the severity and impact of comorbid conditions (Sangha et al., 2003). The SCQ was previously shown to have modest correlations with resource utilization and health status one year later and moderately strong associations with data acquired from medical records (Sangha et al., 2003). Participants respond with a yes (1 point) or no (0 points) to 13 different health conditions, whether they are receiving treatment, and whether the condition limits activities. There is an additional option for participants to enter two other diagnoses that were not included in the questionnaire. Total scores for the SCQ can range from 0 to 45. The measure showed good reliability in our sample (Cronbach's alpha = 0.82).

#### Body compassion

Body compassion was measured using the 23-item Body Compassion Scale (BCS), a newly established reliable and valid self-report measure (Altman et al., 2020). It consists of three subscales: acceptance (5 items), defusion (9 items), and common humanity (9 items). Subscales can be examined individually or combined to produce a total score. Item responses are on a 5-point Likert scale and range from 1 (*Almost Never*) to 5 (*Almost Always*); the defusion subscale is reverse-coded. Higher scores indicate higher body compassion. The total score was used in primary analyses; subscale scores were explored in secondary analyses.

*Acceptance* reflects the intentional embrace of all aspects of the physical body exactly as they are in the present (example item: “I'm tolerant of my body's flaws and inadequacies.”). *Defusion* is relating to one's body from the stance of an observer, as opposed to over-identification with the body as the self [example item (reverse scored): “When my body isn't responding the way I want it to, I tend to be tough on myself”]. *Common humanity* underscores that physical pain, injury, and sickness are part of a collective, universal human experience (example item: “When I feel frustrated with my body's inability to do something, I try to remind myself that most people in my condition feel this way at some point”) (Altman et al., 2020). The measure showed excellent reliability in our sample (Zimmaro et al., 2024) (Cronbach's alpha: total score α = 0.94, acceptance α = 0.90, common humanity α = 0.95, defusion α = 0.91).

#### Mindfulness

Mindfulness was quantified using the Five Facet Mindfulness Questionnaire-15 item (FFMQ-15), which is a reliable and valid measure of mindfulness (Baer et al., 2008; Gu et al., 2016). The 15-item scale is comprised of five mindfulness subscales: observing, describing, acting with awareness, non-judging, and non-reactivity (Baer et al., 2008; Gu et al., 2016; Baer et al., 2006). Item responses range from 1 (*Never or very rarely true*) to 5 (*Very often or always true*), with higher scores reflecting greater mindfulness. Primary analyses were conducted using the total score, and subscales were used in exploratory secondary analyses. The measure showed good to acceptable reliability in our sample (Cronbach's alpha: total score α = 0.84, observing α = 0.59, describing α = 0.78, acting with awareness α = 0.75, non-judging α = 0.72, non-reactivity α = 0.603).

#### Distress

Distress was measured using the 14-item Hospital Anxiety and Depression Scale (HADS) (Zigmond and Snaith, 1983), which has been widely used among diverse cancer samples (Bjelland et al., 2002; Moorey et al., 1991; Vodermaier and Millman, 2011; Walker et al., 2007). Items responses range from 0 to 3, with higher scores reflecting higher distress. The questionnaire includes subscales measuring depression and anxiety, and the combined total score was used as a measure of overall distress. The measure showed excellent reliability in our sample (Cronbach's alpha: total score α = 0.90).

### Statistical analyses

First, a table was created summarizing cohort characteristics using counts and percentages for categorical variables and using the mean, standard deviation, and range (minimum, maximum scores) for continuous variables. Plots were created to explore the relationships between HADS, BCS, FFMQ, and their subscales. To assess the relationships between variables, Pearson's correlation coefficients were calculated, with Holm correction applied to adjust for multiple comparisons.

Second, to explore the effect of body compassion (BCS) in the relationship of mindfulness (FFMQ-15) on distress (HADS), we employed linear regression models following Baron and Kenny's method (Baron and Kenny, 1986) and calculated the bootstrapped mediation effect (Preacher and Hayes, 2004). All models controlled for theoretically-driven covariates of age at diagnosis, metastatic disease status, and current treatment status.

Finally, to explore potential moderators of disease burden (e.g., metastatic disease status, current treatment status, current ostomy status, and total medical comorbidities), individual linear regression models were constructed for each moderating variable of interest, including its interaction with body compassion. To further identify the best-fitting model of distress, least absolute shrinkage and selection operator (LASSO) regression was used to construct a complete case model incorporating the terms of interest. The optimal set of variables was determined by identifying the value of λ that minimized error during 10-fold cross-validation, resulting in the best predictive model. To further refine the final model and reduce noise, variables were selected based on a marginal false discovery rate below 0.1, provided they also maintained a low cross-validation error. *P*-values were calculated based on a null hypothesis of no effect against a two-sided alternative at a significance threshold of 0.05. Analysis was performed using R version 4.3.1 (The R Foundation for Statistical Computing). LASSO was performed utilizing the ncvreg R package [v3.14.1] (Breheny and Huang, 2011).

## Results

### Sample characteristics

The study sample has been previously described (Zimmaro et al., 2024), but will be summarized in brief here, consisted of 54 individuals diagnosed with CRC. The sample was mostly female (61%) and not in current treatment (83%). About a quarter of the sample had metastatic disease, had a current or previous ostomy appliance, and were diagnosed with rectal (vs. colon) cancer. Table 1 shows sample demographic, medical, and psychosocial characteristics.

**Table 1:** Participant Characteristics (*N* = 54).

**Characteristics**	**Mean (SD, range) or frequency % (*n*)**
**Demographic variables**	
Age	*M =* 64.7 (SD = 11.7, range = 32–90)
**Gender**	
Female	61% (33)
Male	39% (21)
**Race**	
White/Caucasian	91% (49)
Black/African American	9% (5)
**Partnered status**	
Married/partnered	70% (38)
Widowed (no partner)	13% (7)
Divorced (no partner)	11% (6)
Single (never married)	6% (3)
**Highest education**	
Graduate degree	26% (14)
College degree	20% (11)
Some college	28% (15)
High school/GED	26% (14)
**Medical variables**	
Age at diagnosis	*M =* 61.5 (SD = 11.6, range = 28–85)
Years since diagnosis	*M =* 3.2 (SD = 1.7, range = 1–12)
**Tumor site**	
Colon	73% (41)
Rectal	24% (13)
**Tumor side**	
Right/bilateral	74% (40)
Left	26% (14)
**Stage**	
I	13% (7)
II	30% (16)
III	33% (18)
IV	24% (13)
**Current treatment**	
No	83% (45)
Yes	17% (9)
**Ostomy status**	
Never had	76% (41)
Had previously	13% (7)
Currently have	11% (6)
Medical comorbidities *(SCQ)*	*M =* 5.78 (SD = 4.52, range = 0–19)
**Psychosocial variables**	
Total distress *(HADS)*	*M =* 10.70 (SD = 7.10, range = 1–27)
Anxiety	*M =* 6.37 (SD = 4.14, range = 0–15)
Depression	*M =* 4.28 (SD = 3.83, range = 0–14)
Total body compassion *(BCS)*	*M =* 82.06 (SD = 19.04, range = 41–115)
Acceptance	*M =* 17.93 (SD = 5.44, range = 15–45)
Defusion	*M =* 33.63 (SD = 8.57, range = 5–25)
Common humanity	*M =* 30.50 (SD = 10.00, range = 9–45)
Total mindfulness *(FFMQ-15)*	*M =* 55.65 (SD = 9.47, range = 36–70)
Observing	*M =* 10.41 (SD = 2.87, range = 4–15)
Describing	*M =* 11.20 (SD = 2.67, range = 5–15)
Non-judging	*M =* 12.22 (SD = 2.42, range = 6–15)
Non-reactivity	*M =* 10.72 (SD = 2.85, range = 4–15)
Acting with awareness	*M =* 11.09 (SD = 2.81, range = 5–15)

SCQ, Self-Administered Comorbidity Questionnaire; BCS, Body Compassion Scale; HADS, Hospital Anxiety and Depression Scale; FFMQ-15, Five Facet Mindfulness Questionnaire-15 item.

Sample characteristics have been previously described, but are provided here for completeness. (Zimmaro et al., 2024).

### Associations between mindfulness and body compassion

Mindfulness and body compassion total scores showed a moderately strong positive correlation (*r* = 0.62, *p* < 0.001) (Supplemental Figure A). The subscale correlations showed a similar, but slightly less robust, pattern of moderate relationships (Supplemental Figure B). Particularly strong associations were observed between the mindful non-judgment subscale with body compassion defusion (*r* = 0.70, *p* < 0.05) and with body compassion acceptance (*r* = 0.49, *p* < 0.05). Other correlation coefficients between the FFMQ and BCS subscales were weak to moderate, with most falling between 0.30 and 0.49 (full range: *r* = 0.11 to 0.70).

As expected, inverse relationships between distress and mindfulness (Supplemental Figures C, D) and distress with body compassion (Supplemental Figures E, F)^1^ were observed. The corresponding scatterplots and correlation matrices are presented in the Supplementary materials.

### Role of body compassion in mindfulness-distress model

Multivariable linear regression analysis controlling for age at diagnosis, metastatic disease status, and current treatment status was used to explore the conditional associations between mindfulness, body compassion, and distress (Table 2). Results showed that higher levels of mindfulness were associated with lower levels of distress (total effect: *B* = −0.39, CI_95%_ [−0.56, −0.16], *p* < 0.001). However, this relationship was significantly mediated by body compassion, which showed a statistically significant indirect effect (*B* = −0.21, CI_95%_ [−0.38, −0.08], *p* < 0.001). Furthermore, after controlling for the effect of body compassion, the association between mindfulness and distress was considerably reduced (direct effect: *B* = −0.18, CI_95%_ [−0.39, 0.02], *p* = 0.10). Overall, body compassion accounted for 54% of the total effect on distress (CI_95%_ [0.21, 1], *p* < 0.001), suggesting that, in this cohort, over half of the relationship between mindfulness and distress operated through body compassion.

**Table 2:** Regression analysis exploring the role of body compassion within the mindfulness-distress relationship.

**Dependent variable relationship**	***B***	**β (beta)**	**CI_95%_ for *B***	***p*-value**
ACME (indirect effect: mindfulness on distress, through body compassion)	−0.21	−0.26	−0.38, −0.08	< 0.001
ADE (direct effect: mindfulness on distress, controlling for body compassion)	−0.18	−0.47	−0.39, 0.02	0.10
Total effect (mindfulness on distress)	−0.39	−0.54	−0.56, −0.16	< 0.001
Proportion of effect explained by body compassion	0.54		0.21, 1.1	< 0.001

Table shows multiple linear regression analysis exploring the conditional associations between mindfulness, body compassion, and distress. The dependent variable was distress (HADS total score). Models controlled for age at diagnosis, metastatic disease status, and current treatment status. Results show that body compassion accounts for a significant portion of the relationship between mindfulness and distress.

B, unstandardized regression coefficient; β, standardized regression coefficient; ACME, Average Causal Mediation Effects; ADE, Average Direct Effect; Total Effect, sum of indirect and direct effects; CI_95%_, 95% Confidence Interval.

### Disease burden moderation

No evidence of moderation by disease burden variables was found. Interactions between body compassion and metastatic disease status, current treatment status, current ostomy status, and total medical comorbidities were not statistically significant (Supplemental Table A). The overall best-fitting model of distress was found to include mindfulness (FFMQ-15 total score), body compassion (BCS total score), and total medical comorbidities (SCQ total score) (Table 3).

**Table 3:** Best fitting regression model of distress.

**Independent variables**	***B***	**β (beta)**	**CI_95%_ for *B***	***p*-value**
Mindfulness	−0.20	−0.26	−0.38, −0.01	0.039
Body compassion	−0.15	−0.41	−0.25, −0.06	0.003
Total medical comorbidities	0.37	0.23	0.04, 0.69	0.034

The dependent variable is distress (HADS total score). The table shows the best-fitting multiple linear regression model of distress, which includes mindfulness (FFMQ-15 total score), body compassion (BCS total score), and total medical comorbidities (SCQ total score), as identified using LASSO method.

B, unstandardized regression coefficient; β, standardized regression coefficient; CI_95%_, 95% Confidence Interval.

## Discussion

Our study revealed several key findings. First, as predicted, we observed that a measure of general mindfulness was positively and moderately correlated with a measure of body compassion, suggesting that these concepts are related but distinct. In particular, body compassion and mindfulness appear to be related through the concept of non-judgment. While body compassion involves non-judgment of one's own physical body, mindfulness involves non-judgment of experiences both within and beyond one's physical body. Importantly, this is the first study to report on the relationship between body compassion and mindfulness in a sample of cancer patients. Our findings contrast with the only other known published research on body compassion and mindfulness, which found a considerably smaller correlation between these constructs in a sample of healthy young women (Altman et al., 2020). The distinction between our findings among individuals with cancer and previous findings among healthy individuals warrants further investigation, as it could suggest that the relationship between mindfulness and body compassion differs across populations, health status, or other contextual factors.

The positive correlation we observed between body compassion and mindfulness was expected given that one of the core components of body compassion is, in fact, being mindful of one's body. However, our results enhance our understanding of this relationship by identifying the mindfulness subscale of non-judgment as having the strongest relationship with body compassion, and, in particular with the defusion and acceptance body compassion subscales. The other mindfulness facets showed weak, non-statistically significant relationships to body compassion, again emphasizing the distinction between these two constructs more broadly. Given that mindful non-judgment involves non-critical awareness of internal experiences, the subscale's alignment with body compassion's elements of defusion (disentanglement from negative body-related thoughts) and acceptance (tolerating bodily changes and limitations) is logical. All three of these concepts emphasize a shift from self-judgment to a more non-reactive, open, and kind attitude toward internal experiences, particularly those related to the body. For CRC survivors, who often endorse struggling with self-criticism, shame, and embarrassment (Phung and Fang, 2023), non-judgment may be the most important mindfulness-based feature of body compassion.

Second, and most notably, body compassion helped to explain the well-established link between mindfulness and distress. We observed that when both mindfulness and body compassion were considered together in statistical models of distress, body compassion emerged as the predominant factor, accounting for 54% of the mindfulness-distress relationship. These findings suggest that anxiety and depression symptoms may be more strongly linked to body compassion than to mindfulness, at least in CRC and perhaps other cancer conditions characterized by prominent bodily changes. Furthermore, the findings allude to the possibility that the positive impact of mindfulness on distress could be primarily operating through increased body compassion. However, while the mediation analysis identified a statistically significant indirect effect, the cross-sectional nature of our data limits the ability to determine causal direction due to the violation of the temporal precedence assumption. Future experimental designs are necessary to further explore mechanistic pathways. While body compassion may be associated with both mindfulness and distress through a potential mediating role, alternative explanations cannot be conclusively ruled out. Nevertheless, the results highlight the concept of body compassion as an exciting and valuable new direction for mindfulness-related research and intervention development, especially in oncology.

Third, contrary to expectations, disease factors did not appear to influence the relationship between body compassion and distress in our cohort. Greater body compassion was associated with lower distress, regardless of whether individuals had metastatic disease, were in treatment, had an ostomy, or had more medical comorbidities. While previously published research in this sample found that body compassion was diminished among patients with these medical characteristics (Zimmaro et al., 2024), the present findings highlight the consistent association between body compassion and lower distress levels, irrespective of such medical factors. Findings suggest that body compassion may be a valuable intervention target for all CRC survivors, and not limited to those with higher disease burden. Interestingly, results showing the best-fit model predicting distress highlighted the importance of including medical comorbidities. It is worth considering the bigger picture for CRC patients; perhaps the number of other comorbid medical problems are drivers of distress that are often overlooked.

While our findings illuminate potential avenues for mindfulness and distress intervention research, particularly for individuals with CRC, the role of dispositional mindfulness in coping with specific, body-centered symptoms remains complex among these patients. While dispositional mindfulness is often linked to adaptive coping strategies and outcomes, its relationship to coping with CRC symptoms—such as ostomy-related issues, gastrointestinal symptoms, and peripheral neuropathy—appears nuanced in earlier research. For example, research suggests that higher levels of non-judgmental mindfulness may reduce disgust towards stoma bags but could also lead to avoidance of stoma care (Reynolds et al., 2014). Additionally, the relationship between dispositional mindfulness and types of peripheral neuropathy (motor or sensory) that are commonly experienced among people with CRC appears to evolve over time (Bonhof et al., 2022). For example, at 1-year post-CRC diagnosis, higher dispositional mindfulness was associated with less severe motor (but not sensory) peripheral neuropathy, while at 2-year follow-up, the reverse pattern was observed (mindfulness was associated with sensory, but not motor, peripheral neuropathy) (Bonhof et al., 2022). Furthermore, the association between dispositional mindfulness and depression among CRC survivors may be mediated by cancer threat appraisal and colorectal symptom distress (Chen et al., 2021). This complexity suggests that the innate ability to be mindful in general may not automatically translate into mindful engagement with specific physical challenges, at least in CRC. Much like a broad stage light, general mindfulness may not have the same focused impact as a spotlight. Rather, a targeted approach to applying mindfulness-related skills to disease- and body-specific challenges, such as through body compassion, may offer an avenue to optimize mindfulness effects on CRC-related distress.

The exploration of body compassion in cancer is a new and promising avenue for research and clinical intervention. For example, Sebri et al. (2022, 2023) have incorporated body compassion in the context of addressing body image and mood concerns in breast cancer survivors. In their pilot study, the authors reported that a four-session body compassion-focused telehealth intervention targeting cognitions, behaviors, and emotions related to the body was associated with significant reductions in anxiety, though no significant changes were observed in depression or body image perception (Sebri et al., 2023). As the study did not include a measure of body compassion, the extent to which body compassion is malleable—or whether it may have played a role in anxiety reduction—remains an open question. As such, research examining the potential mediating role of body compassion in shaping distress outcomes is still emerging, and the current study contributes to this growing area by beginning to explore these relationships empirically.

### Clinical implications

In recent years, there has been a rapidly growing interest in adapting mindfulness-based interventions to address specific disease-related challenges (Loucks et al., 2022). The development of mindfulness interventions that emphasize body compassion and incorporate targeted skills to enhance it could offer a novel approach to addressing distress related to CRC. Body-based mindfulness practices, such as body scan meditations, walking meditations, and even loving-kindness meditations focusing on one's physical body may be particularly salient for body compassion due to their focus on fostering a non-judgmental and kind relationship with the physical body. Findings from the present study also suggest that interventions encouraging a compassionate stance toward one's body may benefit CRC survivors regardless of disease burden and at any point in the cancer journey. A recent meta-analysis found that while mindfulness-based stress reduction had no particular effect on reducing distress relating to body image among breast cancer patients, a mindfulness-based intervention that taught mindfulness skills specifically to enhance body appreciation and self-compassion (“My Changed Body”) (Sherman et al., 2018) did reduce body image distress (Gopan et al., 2024). Among people with CRC, however, few studies exist.

### Limitations

Several limitations should be considered. First, the cross-sectional design of this study violates the temporal assumption required for formal mediation analysis. As a result, the findings should be interpreted with caution, as causality cannot be established. However, exploring potential mediation pathways in cross-sectional data still allows for hypothesis generation for guiding future longitudinal studies. Future longitudinal or interventional studies should explore whether body compassion indeed serves as a full mediator of mindfulness on distress. Second, our sample mostly consisted of older, white CRC survivors who had completed treatment and results may not generalize to more diverse samples. Third, this study examines secondary outcomes and may not be adequately powered to detect differences, such as disease burden variables. A small number of participants had a current ostomy or were currently in treatment. Results are exploratory in nature and help to provide directions for future research. These limitations could be addressed in future trials among larger samples of clinical and/or more diverse cancer populations.

### Future directions

Important future directions of this line of research include examining the stability of body compassion over time with no intervention, as well as how sensitive body compassion is to change with intervention. The relationship between the Body Compassion Scale with other mindfulness-based body-specific measures should also be explored, especially as more types of questionnaires are being developed [e.g., Embodied Mindfulness Questionnaire (Khoury et al., 2023), Body Mindfulness Questionnaire (Burg et al., 2017)]. Furthermore, body compassion in populations with chronic disease, chronic pain, cancer, and clinically significant distress should be compared to healthy populations to consider differences. Mindfulness intervention studies could include measures of body compassion to track covarying relationships. More specifically, empirical data is needed to test whether proposed mindful interventions like loving-kindness meditations and body scans significantly affect body compassion. A four-session body compassion intervention has been previously developed and been associated with reductions in anxiety in a pilot study for breast cancer patients (Sebri et al., 2022). A future study could compare a mindfulness intervention arm to a body compassion intervention arm to examine changes in distress in CRC patients. Perhaps this targeted treatment that focuses on compassion towards one's body could support quality of life in CRC survivors.

## Conclusions

In a sample of individuals diagnosed with CRC, body compassion (i.e., regarding one's physical body with a sense of compassion, mindfulness, and common humanity) appeared to be a factor within the mindfulness-distress relationship. While our data supported the well-established relationship between higher mindfulness and lower distress, our results extended this by finding that this relationship could be mostly explained by body compassion. Furthermore, the relationship between body compassion and distress did not vary by several measures of disease burden. While a causal pathway cannot be determined due to the cross-sectional nature of our data, body compassion should be further explored in future studies as a potential mechanistic pathway by which mindfulness helps to reduce distress among individuals diagnosed with cancer.

## Data Availability

The raw data supporting the conclusions of this article will be made available by the authors, without undue reservation.

## Footnotes

^1^Although the association between distress and body compassion was previously reported in our earlier work (Zimmaro et al., 2024), these results are presented again in accordance with the methodological framework outlined by Baron and Kenny (1986) to verify that the necessary statistical assumptions for conducting the mediation analyses in the present study are satisfied.
