# Supplementary material for: Beyond mindfulness: the importance of body compassion in colorectal cancer distress
Source: Front Psychol. 2025 Oct 8;16:1618389. doi: 10.3389/fpsyg.2025.1618389 (PMC12540465; doi:10.3389/fpsyg.2025.1618389)
Supplement: Supplementary file 1 [file Supplementary_file_1.pdf]

Supplemental Table

Supplemental Table A. Regression Models of Body Compassion on Distress with Disease Burden Moderation

	Model 1			Model 2			Model 3			Model 4		
**Independent Variables**	**B**	**$CI_{95\%}$**	**Sig.**	**B**	**$CI_{95\%}$**	**Sig.**	**B**	**$CI_{95\%}$**	**Sig.**	**B**	**$CI_{95\%}$**	**Sig.**
Mindfulness	-0.19	-0.39, 0.00	0.06	-0.16	-0.26, 0.04	.11	-0.17	-0.27, 0.03	0.10	-0.20	-0.38, -0.01	0.04
Body Compassion	-0.21	-0.32, -0.09	<.001	-0.17	-0.27, -0.07	.001	-0.19	-0.29, -0.09	<.001	-0.17	-0.24, -0.01	0.04
Metastatic Disease	-4.7	-19.0, 9.2	0.5									
Body Compassion * Metastatic Disease	0.07	-0.10, 0.25	0.4									
Current Treatment				12	-5.0, 29.0	0.20						
Body Compassion * Current Treatment				-0.13	-0.36, 0.09	0.20						
Current Ostomy							8.1	-13.0, 29.0	0.40			
Body Compassion * Current Ostomy							-0.12	-0.40, 0.17	0.40			
Total Comorbidities										0.09	-1.7, 1.9	>0.9
Body Compassion * Total Comorbidities										0.00	-0.02, 0.03	0.8

Note. This table shows the results of four separate multiple linear regression models exploring whether disease burden variables moderate the relationship between body compassion and distress (HADS total score). No statistically significant moderators were observed. Mindfulness: Five Facet Mindfulness Questionnaire-15 item. Metastatic disease, current treatment, current ostomy statuses: dichotomized yes/no. Total Comorbidities: Self-Administered Comorbidity Questionnaire total score. *Abbreviations:* B = unstandardized regression coefficient. CI = confidence interval. * = interaction assessed between the two variables.

Supplemental Figures

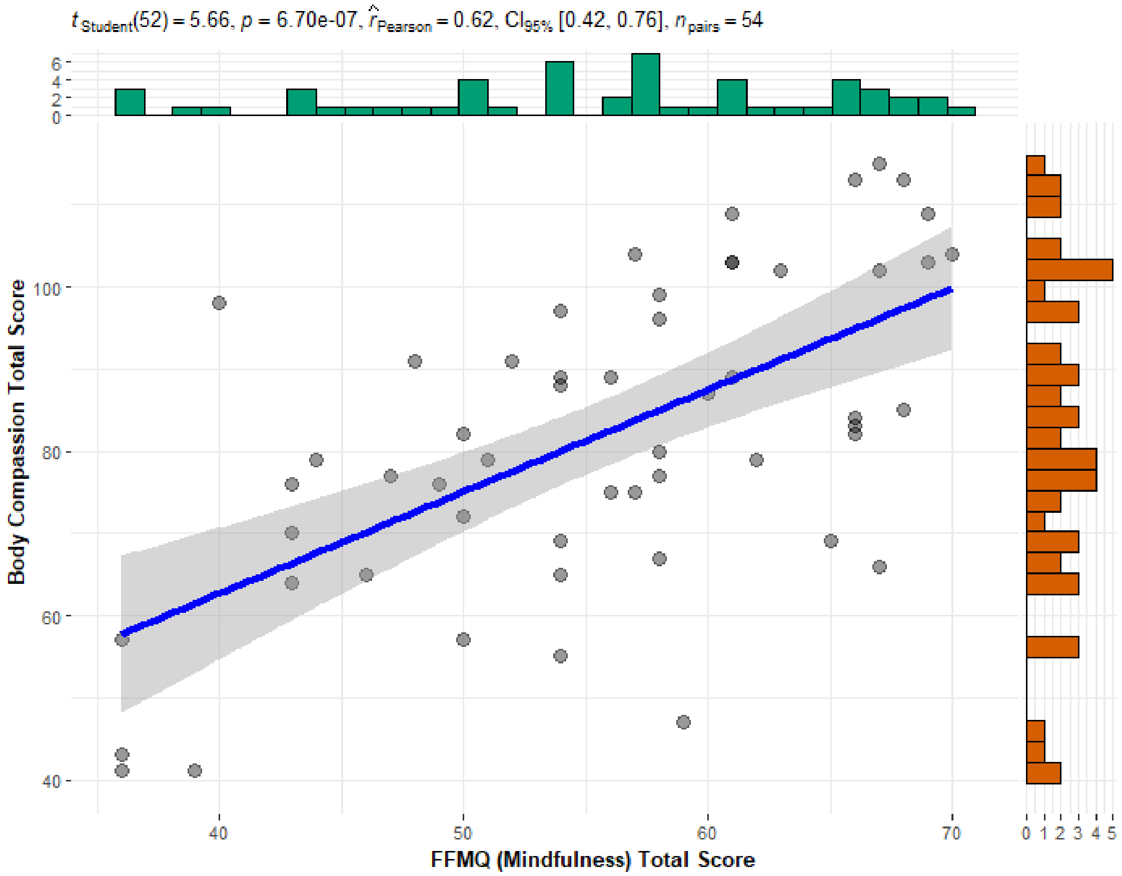

Supplemental Figure A. *Scatterplot of the relationship between body compassion and mindfulness.* The scatterplot shows the linear relationship between body compassion and mindfulness total scores. Pearson's correlation test revealed a statistically significant correlation between the two measures with a medium-to-large effect size ($t_{student}(52) = 5.66$, $p < .001$, $r_{Pearson} = 0.62$, $CI_{95\%}$[0.42, 0.76], N=54 pairs).

Body Compassion Scale \ Five Facet Mindfulness Questionnaire-15 Item	**Total Score**	Observing	Describing	Nonjudging	Non-Reactivity	Acting with Awareness
Acceptance	0.53	~~0.30~~	~~0.39~~	0.49	~~0.36~~	~~0.33~~
Common Humanity	0.47	~~0.34~~	~~0.34~~	~~0.13~~	~~0.40~~	~~0.38~~
Defusion	0.49	~~0.11~~	~~0.33~~	0.70	~~0.32~~	~~0.30~~
**Total Score**	0.62	~~0.31~~	0.44	0.53	0.46	0.43

Supplemental Figure B. *Correlalogram for Body Compassion and Mindfulness***.** The correlalogram shows the Pearson's correlation coefficients for the total score and subscales of the Body Compassion Scale and the Five Facet Mindfulness Questionnaire-15 item. Shading corresponds to the strength of a positive relationship (darker = stronger correlation.) Holm adjustment was applied to p-values to account for multiple comparisons. 'X' indicates a non-statistically significant relationship (p<.05, Adjustment: Holm).

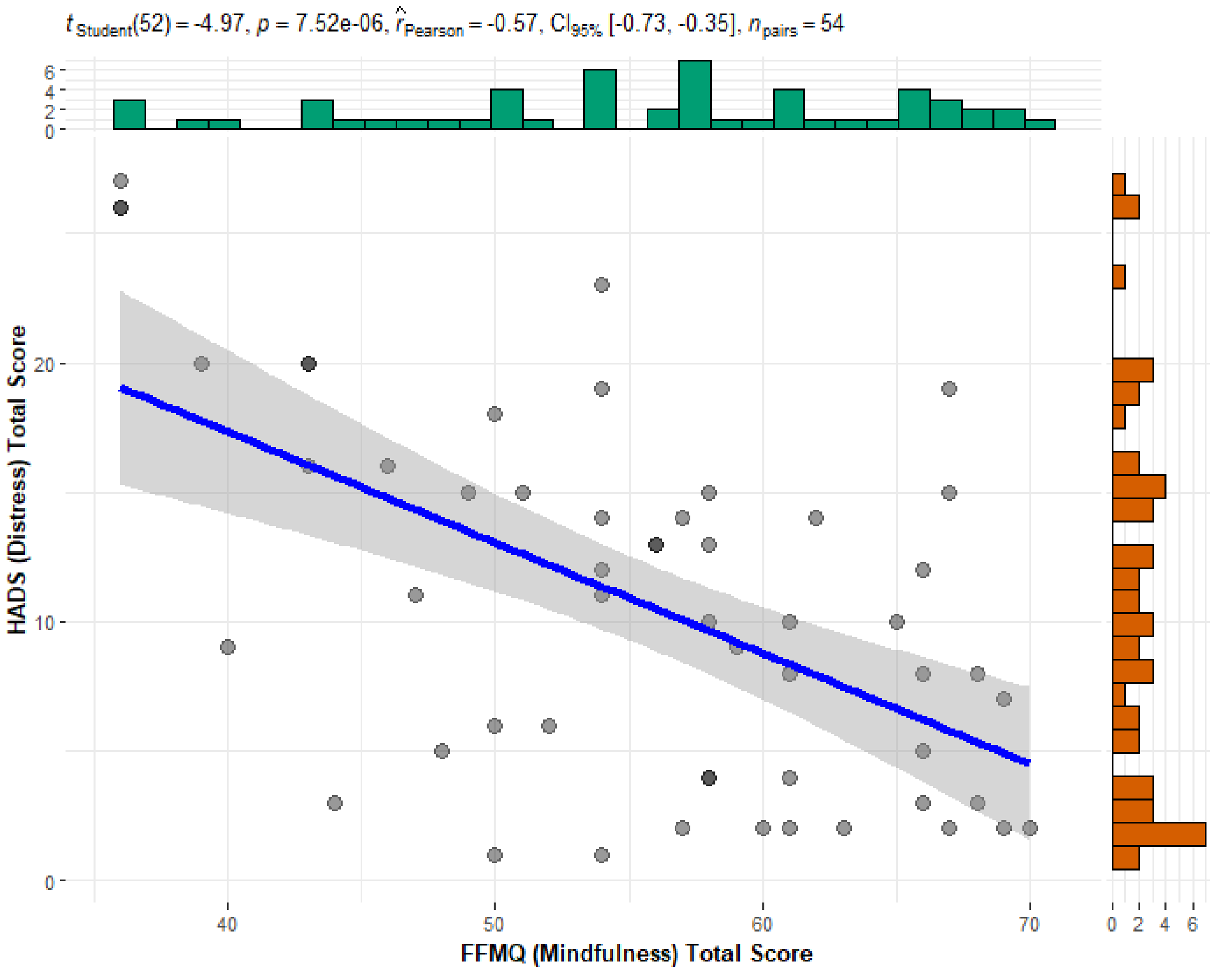

Supplemental Figure C. *Scatterplot of the relationship between mindfulness and distress*. The scatterplot shows the linear relationship between mindfulness (FFMQ-15) and distress (HADS) total scores. Pearson's correlation test revealed a significant negative correlation between the two measures with a medium-to-large effect size ($t_{student}(52) = -4.97$, $p < .001$, $r_{Pearson} = -0.57$, $CI_{95\%}$[-0.73, -0.35], N=54 pairs).

**Hospital Anxiety and Depression Scale**		**Five Facet Mindfulness Questionnaire-15 Item**					
		**Total Score**	Observing	Describing	Nonjudging	Non-Reactivity	Acting with Awareness
	Depression	-0.55	~~-0.18~~	-0.46	-0.49	-0.46	~~-0.36~~
	Anxiety	-0.47	~~-0.17~~	~~-0.33~~	-0.54	~~-0.24~~	~~-0.38~~
	**Total Score**	-0.57	~~-0.20~~	-0.44	-0.58	~~-0.38~~	-0.41

Supplemental Figure D. *Correlalogram of mindfulness and distress*. The correlalogram shows the Pearson's correlation coefficients for the total score and subscales of the Hospital Anxiety and Depression Scale and the Five Facet Mindfulness Questionnaire-15 item. Shading corresponds to the strength of the negative relationship (darker = stronger negative correlation.) Holm adjustment was applied to p-values to account for multiple comparisons. 'X' indicates a non-statistically significant relationship (p<.05, Adjustment: Holm).

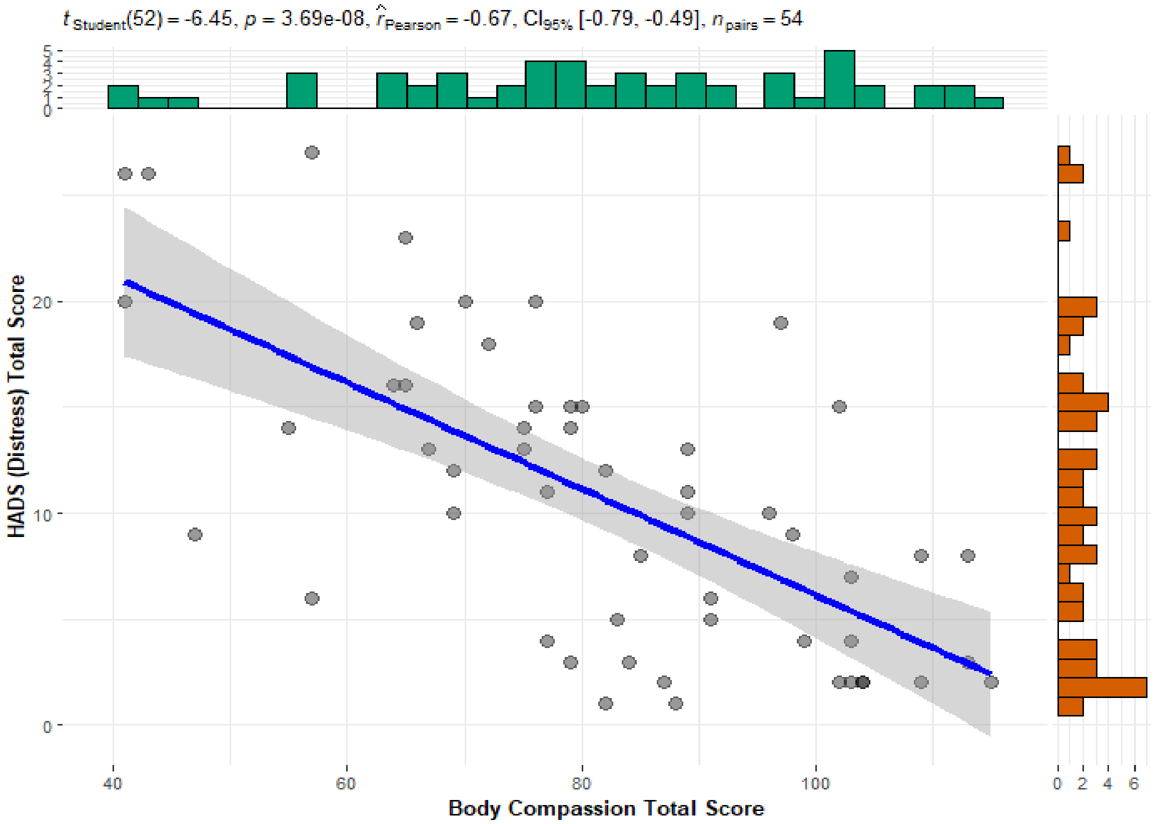

Supplemental Figure E. *Scatterplot of the relationship between body compassion and distress*. The scatterplot shows the linear relationship between body compassion (BCS) and distress (HADS) total scores. Pearson's correlation test revealed a significant negative correlation between the two measures with a medium-to-large effect size ($t_{student}(52) = -6.45$, $p < .001$, $r_{Pearson} = -0.67$, $CI_{95\%}[-0.79, -0.49]$, N=54 pairs).

Hospital Anxiety and Depression Scale					
	Depression	-0.68	-0.57	-0.50	-0.58
	Anxiety	-0.52	-0.56	-0.21	-0.55
	**Total Score**	-0.67	-0.63	-0.39	-0.63
		**Total Score**	Defusion	Common Humanity	Acceptance
		**Body Compassion Scale**			

Supplemental Figure F. *Correlalogram of body compassion and distress*. The correlalogram shows the Pearson's correlation coefficients for the total score and subscales of the Hospital Anxiety and Depression Scale and the Body Compassion Scale. Shading corresponds to the strength of the negative relationship (darker = stronger negative correlation.) Holm adjustment was applied to p-values to account for multiple comparisons. 'X' indicates a non-statistically significant relationship (p<.05, Adjustment: Holm).
